# Could symptom overlap of COVID-19 and Guillain–Barré syndrome mask an epidemiological association?

**DOI:** 10.1007/s00415-021-10515-8

**Published:** 2021-03-17

**Authors:** Martin K. R. Svačina, Felix Kohle, Alina Sprenger, Helmar C. Lehmann

**Affiliations:** Department of Neurology, Faculty of Medicine, University Hospital of Cologne, Kerpener Straße 62, 50937 Köln, Germany

Dear Sirs,

Whether COVID-19 can trigger Guillain–Barré syndrome (GBS) is currently controversially discussed. Recent studies in this and other journals [2–4] indicate an association. However others did not find any epidemiological evidence so far [1]. In this latter study, no significant association between COVID-19 and GBS was found. In 47 patients with GBS (of those 13 patients with definite, 12 with probable, and 22 patients without COVID-19) there was no atypical pattern in incidence, clinical presentation or response to established therapies in COVID-19 associated GBS [1]. Uniquely, a higher rate of assisted ventilation requirement was observed in GBS subsequent to COVID-19 [1].

Notably, several case series already conveyed a rapid sequence and even significant overlap of COVID-19 pneumonia and GBS [4–6]. We also observed such a rapid temporal sequence in three patients consecutively treated in our department between October 2020 and January 2021:

The first patient, a 76-year-old male, presented with a reduction of his general condition and dyspnoea. Thoracic CT revealed pneumonic infiltrates typical for COVID-19. PCR analysis for SARS-CoV-2 were equivocal. Four days after hospitalization, the patient showed a rapidly evolving flaccid tetraparesis with general areflexia and phrenicobulbar involvement with consecutive requirement of intensive care. Cerebrospinal fluid (CSF) showed albuminocytologic dissociation and IgM autoantibodies against sulfatide were detected in serum (Table 1). Nerve conduction studies (NCS) revealed an axonal-demyelinating sensorimotor polyradiculoneuropathy. Intravenous immunoglobulin (IVIg) led to slight improvement of motor symptoms, with persistence of flaccid tetraparesis. The second patient, a 53-year-old male presenting with acute left-side facial palsy, tongue deviation and right-side oculomotor palsy, reported rhinorrhea with onset the day before and had contact to a COVID-19 patient four days prior to hospitalization. PCR analysis was positive for SARS-CoV-2 and thoracic CT showed pulmonary infiltrates typical for COVID-19. The following day symptoms progressed to severe dysphagia and dysarthrophonia. CSF showed albuminocytologic dissociation, without serological proof of ganglioside autoantibodies. NCS revealed absent left orbicularis oculi reflex and mild demyelinating sensorimotor polyradiculoneuropathy with proximal conduction blocks. COVID-19 associated polyneuritis cranialis was suspected and treatment with remdesivir (for 5 days), IVIg (0.4 g/kg for 5 days) and dexamethasone (6 mg for 10 days), led to almost full recovery. The third patient, a 68-year-old female, presented with a rapid progressive paraparesis deteriorating to flaccid tetraparesis with dyspnoea. NCS revealed severe demyelinating polyradiculoneuropathy and albuminocytologic dissociation was present in CSF examination. PCR analysis was positive for SARS-CoV-2. Thoracic CT revealed pulmonary infiltrates typical for COVID-19. Due to rapid deterioration, intensive care was necessary and IVIg (2 g/kg) was administered over 5 days leading to only mild improvement of paresis and dyspnoea. Subsequent PCR testings for SARS-CoV-2 remained negative. Eighteen days after hospitalization the patient showed persisting severe flaccid tetraparesis.

**Table 1:** Characteristics of three COVID-19 related cases of Guillain–Barré syndrome and variants

	Case 1	Case 2	Case 3
Age	76	53	68
Sex	Male	Male	Female
GBS symptoms	Tetraparesis, areflexia	Facial palsy, oculomotor palsy, hypoglossal palsy	Tetraparesis, areflexia, distal paraesthesia
NCS pattern	Axonal-demyelinating sensorimotor	Demyelinating sensorimotor, vanished orbicularis oculi reflex	Axonal-demyelinating sensorimotor
CSF	Albuminocytologic dissociation	Albuminocytologic dissociation	Albuminocytologic dissociation
Ganglioside autoantibodies	Anti-sulfatide IgM (unspecific)	None	None
Therapy	IVIg (0.4 g/kg)	IVIg (0.4 g/kg), dexamethasone (6 mg), (remdesivir [10 days])	High dose IVIg (2 g/kg)
Outcome (at discharge)	Severe tetraparesis	Mild oculomotor palsy	Severe tetraparesis
Sars-CoV-2 PCR	Equivocal	Positive	Positive
Sars-CoV-2 symptoms (overlapping with neuropathy)	Atypical pneumonia	Atypical pneumonia, rhinorrhoea, headache	Dyspnoea

The first and the third patient fulfilled Brighton level 1 diagnostic criteria for GBS [7], the second patient showed a GBS variant with predominant cranial nerve involvement [4]. Alternatively, a direct viral infection of cranial nerves could not be surely excluded in the latter mentioned case. Two patients suffered from definite and one patient suffered from probable COVID-19 in accordance to ECDC criteria (European centre for disease prevention and control) [8]. Unspecific anti-sulfatide IgM was detected in one patient underlining the observation of specific anti-ganglioside antibodies to be uncommon in COVID-19 related GBS [3, 4]. Furthermore, all three patients showed a rapid temporal sequence between COVID-19 and GBS onset. All patients required intensive care with two patients remaining severely affected after immunomodulatory treatment.

Prototypic GBS usually arises two to four weeks after an infection [9–11]. We would like to stress that a rather rapid, often parainfectious temporal evolution of COVID-19 and severe symptoms of GBS could be a specific feature of COVID-19 related GBS [4]. This is important since it may increase the likelihood of missing the diagnosis of GBS in ventilated COVID-19 patients that are not examined by neurologists and not accessible to standard diagnostic tests due to hygiene restrictions. On the other hand the asymptomatic phase of SARS-CoV-2 infection that can last up to 14 days has to be considered [12]. Lastly, it cannot be excluded that COVID-19 related neuropathy may be a specific clinical condition sharing features of GBS but may present with other clinical symptoms and diagnostic tests that cannot be substantiated by the rather small number of 25 GBS cases associated with definite or probable COVID-19 as reported by Keddie and colleagues [1]. In our case series, regarding a certain clinical amelioration of all patients after IVIg infusion, COVID-19 related GBS appears more likely than a direct infection of peripheral nerves with COVID-19. Additional prospective epidemiological studies with rigorous case ascertainment are necessary to further gain insights into COVID-19 related GBS.

## Data Availability

The data that support the findings of the presented cases are available on request from the corresponding author. The data are not publicly available due to privacy or ethical restrictions.
